# Within-Host Dynamics of Multi-Species Infections: Facilitation, Competition and Virulence

**DOI:** 10.1371/journal.pone.0038730

**Published:** 2012-06-21

**Authors:** Sandeepa M. Eswarappa, Sylvie Estrela, Sam P. Brown

**Affiliations:** 1 Department of Cell Biology, Lerner Research Institute, Cleveland Clinic, Cleveland, Ohio, United States of America; 2 Centre for Immunity, Infection and Evolution, University of Edinburgh, Edinburgh, United Kingdom; 3 Institute of Evolutionary Biology, School of Biological Sciences, University of Edinburgh, Edinburgh, United Kingdom; 4 Department of Zoology, University of Oxford, Oxford, United Kingdom; Centro de Investigación y de Estudios Avanzados, Mexico

## Abstract

Host individuals are often infected with more than one parasite species (parasites defined broadly, to include viruses and bacteria). Yet, research in infection biology is dominated by studies on single-parasite infections. A focus on single-parasite infections is justified if the interactions among parasites are additive, however increasing evidence points to non-additive interactions being the norm. Here we review this evidence and theoretically explore the implications of non-additive interactions between co-infecting parasites. We use classic Lotka-Volterra two-species competition equations to investigate the within-host dynamical consequences of various mixes of competition and facilitation between a pair of co-infecting species. We then consider the implications of these dynamics for the virulence (damage to host) of co-infections and consequent evolution of parasite strategies of exploitation. We find that whereas one-way facilitation poses some increased virulence risk, reciprocal facilitation presents a qualitatively distinct destabilization of within-host dynamics and the greatest risk of severe disease.

## Introduction

Parasitism is ubiquitous – all cellular organisms are potential hosts to damaging infectious agents, from viruses to worms. Parasites (organisms that live on or in a host and get their food from or at the expense of its host) are now recognized as dominant components of diverse biological communities, both in terms of diversity and even in terms of total biomass [1]. Given the incredible prevalence and diversity of parasites within host populations, it is unsurprising that host individuals are often found to be co-infected with multiple parasite species [2]. However, research into host-parasite interactions remains dominated by the study of single infections in isolation, with only occasional consideration for the mechanistic interactions between parasites and their ecological and evolutionary implications [3], [4], [5], [6], [7], [8]. Pedersen and Fenton categorized a range of mechanisms that can cause parasite interactions, ranging from reciprocal competition (e.g. species A and species B compete for a shared resource, thus A inhibits the growth of B and vice-versa) to reciprocal facilitation (e.g. species A and species B cross-feed on the byproducts of their partner, thus A enhances the growth of B and vice-versa) [3].

Studying multi-species infections is of particular biomedical importance as several infectious diseases are complicated by secondary or opportunistic infections, for example, HIV and associated infections (such as tuberculosis) [9], [10], and lyme disease and its associated tick-born infections [11]. Besides impeding host recovery [12], co-infections can create confusion and delay in diagnosis and treatment.

Over the past few years there has been an increasing interest in studying multispecies co-infections [13], [14]. A recent study in wild-rodent populations demonstrated that host susceptibility to a microparasite infection was significantly affected by secondary infections [14]. Their results also highlighted the possibility of different types of microparasite species associations leading to different types of interactions (one way/reciprocal positive/negative association). For example, while infection with the bacterium *Anaplasma phagocytophilum* decreased the host susceptibility to *Bartonella spp.* infection, *Anaplasma phagocytophilum* increased susceptibility to cowpox virus. According to another study, host susceptibility to *Streptococcus pneumonia* transmission and disease are increased if previously infected with influenza [15]. The negative influence of co-infection on mortality was highlighted in a study on rainbow trout showing that fish co-infection with an ectoparasite and a bacterial pathogen significantly decreased the fish survival. Although mono-infections with the ectoparasite did not affect fish survival, it enhanced the susceptibility to the bacterial pathogen [16].

Here we use basic ecological theory to investigate the within-host dynamical consequences of various mixes of competition and facilitation between a pair of co-infecting species. We then consider the implications of these dynamics for the virulence of co-infections and consequent evolution of parasite strategies of exploitation. We find that whereas one-way facilitation poses some increased virulence risk, reciprocal facilitation presents a qualitatively distinct destabilization of within-host dynamics and the greatest risk of severe disease.

## Materials and Methods

To describe the growth of two parasite species (A and B) within a single host, we begin with the classic Lotka-Volterra two species competition equations [17].
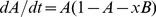
(1a)

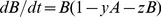
(1b)Here, *A* and *B* represent densities of A and B, respectively, scaled to the carrying capacity of A (for details on the rescaling of model 1, see [17] and Text S1). *x* and *y* are interspecific competition coefficients, measuring the relative competitive (inhibitory) weight of an interspecific individual, relative to a conspecific individual. Finally, *z* is a measure of intraspecific competition within the B population (implying that the carrying capacity of B is 1/*z* times that of A, where *z*>0). These assumptions imply that single species infections will tend to stable equilibrium densities (A* = 1 for species A, B* = 1/*z* for species B), i.e., we describe the dynamics of chronic infections [18]. This assumption of single species chronicity can be viewed as a statement that on the timescale of superinfection (expected time from 1^st^ to 2^nd^ infection), the initial infection dynamics are relatively stable. As infection dynamics become more acute, then the incidence of superinfection will correspondingly decrease (in other words, the multiple-infection issues addressed in this paper are generally a property of parasites that are relatively chronic).

In the classic implementation of Model 1, all parameters are constrained to be positive (i.e., we have both intraspecific and interspecific competition). However, if we allow *x* and/or *y* to turn negative, we can consider the potential for reciprocal or one-way facilitation [17]. Specifically, if *x<*0, parasite B will facilitate the growth of A, and if *y<*0, parasite A will facilitate the growth of B.

To link the within host dynamics of A and B to virulence (additional mortality and/or morbidity), we assume that virulence *V*(*t*) is proportional to the densities of the two parasites [19], hence *V*(*t*) = *a A*(*t*)+*b B*(*t*).

**Figure 1:**
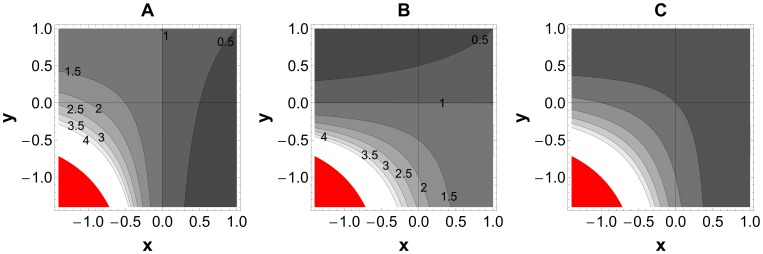
Effect of various mixes of facilitation and competition on the within-host dynamics of coinfecting species. A, Equilibrium densities of parasite A (A*). **B,** Equilibrium densities of parasite B (B*). **C,** Virulence such that *V* = aA*+bB*. The red region represents the region where all equilibria are destabilized (i.e. *xy>z*, *x*<0 and *y*<0). Darker regions indicate lower values. The values on the contour lines indicate the relative densities of parasite at equilibrium. The line *x* = 0 defines the density of parasite A alone, and *y* = 0 defines the density of parasite B alone. The parameter values used are *a* = *b* = *z* = 1.

## Results and Discussion

To characterize the dynamics of Model 1, we first note that the system has 3 non-zero equilibria – either we find A alone (at A* = 1), B alone (at B* = 1/*z*) or A plus B coexistence (at A* = (*z−x*)/(*z−xy*) and B* = (1*−y*)/(*z−xy*)). A stability analysis of these equilibria [17], [20] reveals that when *y>*1 (i.e., if A is strongly inhibitory to B) then A alone is stable. Similarly, if *x*>*z* (i.e., if B is strongly inhibitory to A), then B alone is stable. If both of these inequalities hold (and therefore interspecific competition dominates) then both A alone and B alone are locally stable (bistable dynamics), with A dominating whenever the proportion of A exceeds (*z−x*)*/*(*z−x−y+1*) (i.e., A*/(A*+B*)). Bistable dynamics describe a simple resident advantage – whichever parasite establishes first is likely to resist colonization and replacement by a novel intruder (Figure S1).

In the absence of strong interspecific competition (i.e., when *x*<*z* and *y*<1), neither species can exclude the other, and so we observe coexistence. In addition, in the absence of facilitation (such that 0<*x<z* and 0<*y*<1) we find that the coexistence equilibrium is stable. Figure 1 illustrates the behaviour of the coexistence equilibrium over the range of parameter values where coexistence is guaranteed. This coexistence remains stable if we introduce one-way facilitation (either *x* or *y* turning negative) and even for weak reciprocal facilitation. However, for sufficiently strong reciprocal facilitation (*x*<0, *y*<0 and *xy>z*) all equilibria are destabilized and the within-host dynamics enter into a runaway process, characterized by uncontrolled growth of both parasite lineages (Red region in Figure 1).

**Figure 2:**
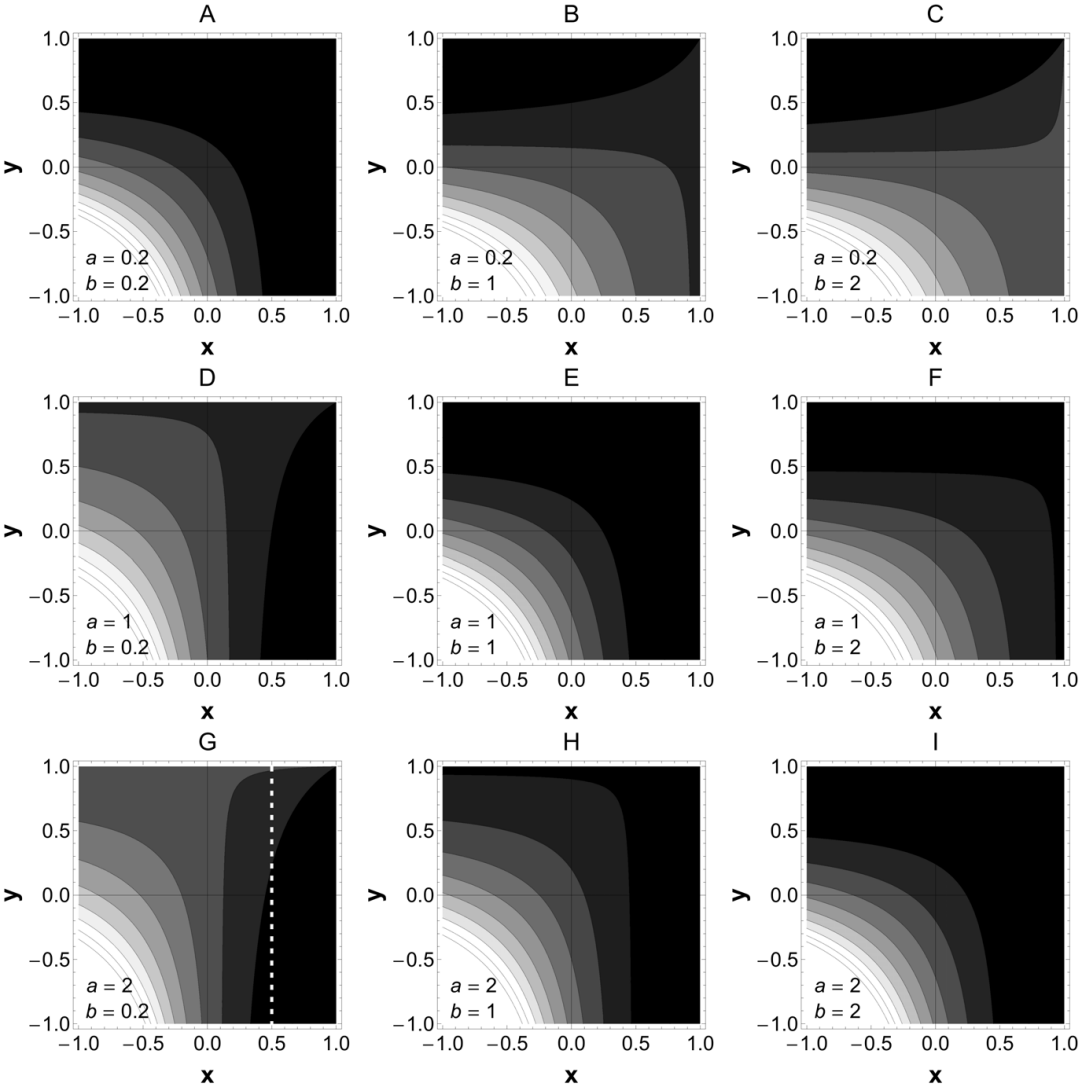
Effect of asymmetric (*a ≠ b*) and symmetric (*a  =  b*) parasites’ contribution to total virulence (V). Given a pair of values (*a, b*) the contour lines in each figure represent the total virulence on the host (V = aA*+bB*) for different values of *x* (parasite B competition/facilitation of parasite A) and *y* (parasite A competition/facilitation of parasite B). Lighter the region higher the virulence. *z* = 1 (i.e., symmetric intraspecific competition). The dashed white line exemplifies a situation where virulence is largely defined by parasite A (a>>b) and parasite B inhibits parasite A (*x*>0). Moving along this line by increasing −*y* (i.e. increasing facilitation to B) may decrease the overall virulence.

Broadly, virulence will tend to increase as *x* and *y* decrease and become negative (as facilitation dominates) (Figure 1C and Table S1). However, if virulence is determined primarily by one or the other of the species (and the other is relatively cryptic with respect to the host) then increasing one-way facilitation can in some cases decrease virulence (Figure 2). For example, if virulence is largely defined by A (*a>>b*) and B inhibits A (*x>*0), then increasing facilitation of B (increasing *–y*) can reduce virulence (see dashed white line on Figure 2G for an example). Figure 2 illustrates that virulence may decrease under one of the following scenarios: a) Increasing reciprocal competition, if virulence of the two parasites is symmetric (*a = b*, see Figure 2A, E, I); b) Increasing competition imposed by the less virulent species on the more virulent species, if there is reciprocal competition (*x*>0 and *y*>0, see Figure 2C, G); c) Increasing facilitation by the more virulent species on the less virulent strain when one-way facilitation (either *x* or *y* negative, e.g. on Figure 2C, G). These results follow from the simple effect that giving aid to (or harming) a competitor acts to increase (or decrease) competitive costs. For illustrative purpose we used a linear mapping between virulence and parasite densities in Figure 1C and 2 (i.e. *V* = aA*+bB*). Relaxing this assumption will change the contour spacings represented in these figures, however the primary prediction of a qualitative shift in virulence given reciprocal facilitation holds for any case where *V* is a monotonically increasing function of *A* and of *B.* Under this more general condition, any run-away in A and B densities will translate to an unbounded increase in virulence.

The various mixes of net facilitation and competition outlined in Figures 1 and 2 provide a simple sketch of more complex within-host interactions, including indirect interactions via inducible (immune-mediated) defences [21] or shared phages [22]. For example, if parasite A suppresses host immunity, that may favour infection by parasite B resulting in a net indirect facilitation of B by A. Similarly, a parasite which induces a host generalised immune response can result in indirect harm to other co-infecting parasites. Note that a more mechanistic predator-prey model has been applied to understand immune-mediated within-host interspecies parasite interactions [21]. While this model focuses explicitly on parasite interactions that are mediated by the host’s immune response (an indirect interaction), our model is more general by assuming both direct and indirect interactions of any net sign.

Reciprocal competition (*x>*0 and *y*>0) can be considered the default net interaction – co-infecting parasites are competing for the limited resource of one single host. However many examples of facilitation can be found in the literature. HIV and oral candidiasis is potentially a good example of one-way facilitation [23]. *Candida albicans,* the fungus that causes oral candidiasis, is a commensal in the normal human oral mucosa. During HIV infection, immunosuppression promotes the proliferation of this fungus beyond normal limits leading to oral candidiasis, thus HIV facilitates the growth of the fungus (if HIV  =  parasite A, then *y*<0). In return, there is no evidence that the enhanced proliferation of *C. albicans* has any marked impact on HIV proliferation, indicating that *C. albicans* remains a commensal towards HIV (*x* close to zero), even as it turns pathogenic towards the shared host (increasing B).

On the other hand, if the facilitation is two-sided (i.e., *x*<0 and *y*<0) the equilibrium densities of both parasites will be higher given co-infection (Figure 1A and B). Of particular concern is the case where the reciprocal facilitation is sufficiently strong to destabilize the coexistence state (i.e., *xy>z*, red region in Figure 1). When this condition is met, the infection is predicted to grow without bounds demanding immediate and rigorous management. Coinfection of HIV and *Mycobacterium tuberculosis* is a potential example for such a dangerous collaboration. HIV not only helps reactivation of dormant *Mycobacterium* bacilli, but also promotes fresh infection and reinfection [24]. Specifically, HIV aids the survival and proliferation of *Mycobacterium* by decreasing the number of CD4 T cells, inactivating macrophage functions and affecting Mycobacterium-specific T cell response [25]. *Mycobacterium* on the other hand boosts the replication of HIV by some unclear mechanism [26]. It has been demonstrated that *Mycobacterium* can increase HIV transcription in transiently transfected T and monocytic cell lines and that *Mycobacterium* increases HIV production in chronically infected or acutely infected monocytic cell lines. A correlation between *Mycobacterium*-induced HIV production and secretion of certain inflammatory cytokines has also been observed [27], [28], [29].

Facilitatory interactions involving HIV are relatively well documented due to the immuno-suppressive impact of HIV and the extent of research effort into this disease. However other examples exist, for instance co-infections of *Salmonella* and *Plasmodium* are suggestive of reciprocal facilitation. Leucopenia during typhoid fever [30] caused by *Salmonella* can facilitate the entry and survival of *Plasmodium* in blood. On the other hand, iron released during RBC lysis in malaria caused by *Plasmodium* can boost the growth of intracellular *Salmonella* [31], [32], [33]. Thus, the combination of typhoid fever and malaria in the same host is a dangerous condition demanding rigorous management. In fact, coinfection of *Salmonella* and *Plasmodium* has been reported in several places across the globe [34], [35], [36], [37], [38]. It is likely that an increasing knowledge of the pathobiology of combination infections will lead to the discovery of many more potentially dangerous collaborations among pathogenic microbes.

Our dynamical analysis of two-species interaction highlights that the dynamics of a focal species can be significantly modulated as a result of mechanistic interactions with a second, co-infecting species (Figure 1 and 2): the equilibrium density of the focal species can be increased, decreased or entirely destabilised as a result of the interaction. These effects raise an important evolutionary question – does selection favour facilitatory or inhibitory (competitive) interactions with co-infecting species (i.e., changes in parasite traits underlying the interspecific interaction parameters *x* and *y*)? An important ingredient in any answer to this question is an understanding of the frequency of coinfection between focal and partner species. If coinfection (with any partner) is a relatively rare occurrence, then standard virulence evolution theory predicts selection will favour intermediate levels of ‘prudent’ exploitation that efficiently balance the advantages of exploitation (transmission) with the costs (host death) [18], [39], [40], [41]. The addition of a second partner co-infection would then induce a non-adaptive perturbation, no matter whether the direction of the effect was towards higher or lower rates of within-host growth (facilitation or competition).

If, in contrast, co-infection is a common and predictable occurrence, then selection could act to modify the single species exploitation strategy given the expected sign of interaction with the partner species. The impact of within-host competition (positive *x* and *y*) on the evolution of virulence has been the subject of a diverse range of models and empirical tests, offering contrasting explanations for either an increase or a decrease in virulence as within-host diversity increases [42]. The different virulence outcomes result from selection of different mechanisms of winning a greater share of the limited host resource – increased within-host replication [39], [43]; decreased contribution to collective exploitation [44], [45]; increased investment in interference competition [22], [46], [47].

The literature on virulence evolution in mixed infections has focused almost entirely on single species interactions among strains that compete largely symmetrically for shared limited resources. What happens when we move away from this single species paradigm? A few studies have considered multi-species competitive interactions and the greater competitive asymmetries that result [48], [49], [50] however to our knowledge there has been no consideration of virulence evolution given facilitatory within-host interactions despite the existence of numerous empirical examples, as detailed above. We propose that repeated facilitatory interactions will select for strategies that maximize a focal species yield in the context of the predictable facilitatory perturbation from the partner species. Specifically, this may take the form of a reduced growth rate, given the expectation of facilitation restoring growth towards the prudent optimum. Under this scenario, facilitatory interactions could form part of a truly mutualistic partnership, in so far as they restored the partner dynamics towards their optima. However, a dependence on a corrective input from a partner species would leave open the possibility of even greater perturbations in the event of the establishment of an inappropriate partnership. For species facing significant uncertainty over the sign of interaction with partner species, a possible solution is to adapt plastic responses, modulating behaviours in response to changes in co-infection status [51], [52].

In addition to the evolutionary context, a further and marked simplification of our model is our limitation to a two-species context. In practice, within-host parasite community structure can be vastly more complex and multi-dimensional, featuring networks of facilitatory and inhibitory interactions. The exploration of appropriately multi-dimensional community models represents an important challenge for future research. Our results hint that networks characterized by reciprocal facilitation will be significantly more prone to extinction (via host death), therefore biasing observed networks towards more robust inhibitory interactions, where the sum of parasite effects is significantly less than their effects alone.

## Supporting Information

Figure S1 **Bistable dynamics of the co-infection (either parasite A alone or parasite B alone at equilibrium).** **A,** Temporal dynamics of the proportion of parasite A (*p  =  A/(A + B)*) for different initial *p* values ranging from 0.1 to 0.9. *y*  = 1.2, *x*  = 0.9, and *z*  = 0.7. The repellor value is at p* = A*/(A*+B*) = (*z−x*)*/*(*z−x−y+1*)  = 0.5 (dashed line). **B,** The threshold of invasion by parasite A (i.e. minimum *p* value for which A invades) increases with *x* and decreases with *y* (*z*  = 1). **C,** The threshold of invasion by parasite A (i.e. minimum *p* value for which A invades) increases with *x* and decreases with *z* (*y*  = 1.2).
(EPS)

Table S1 Effect of increasing *x*, *y*, and *z*, on the densities of A*, B*, and on total virulence (V*) at stable coexistence (A* ≠ 0 and B* ≠ 0).
(DOC)

Text S1 Model Equilibria and Stability Analysis.
(DOC)
