# Supplementary material for: Within-Host Dynamics of Multi-Species Infections: Facilitation, Competition and Virulence
Source: PLoS One. 2012 Jun 21;7(6):e38730. doi: 10.1371/journal.pone.0038730 (PMC3380906; doi:10.1371/journal.pone.0038730)
Supplement: Table S1 — Effect of increasing x, y, and z, on the densities of A*, B*, and on total virulence (V*) at stable coexistence (A* ≠ 0 and B* ≠ 0). (DOC) [file pone.0038730.s002.doc]

**Table S1. Effect of increasing *x*, *y*, and *z*, on the densities of *A**, *B**, and on total virulence (*V**) at stable coexistence (*A** ≠ 0 and *B** ≠ 0).**

|  | *A** | *B** | *V** |
| --- | --- | --- | --- |
| ***x*** | *Decreases* | *Increases if y > 0* | *Increases if az < by* |
| ***y*** | *Increases if x > 0* | *Decreases* | *Increases if x > b/a* |
| ***z*** | *Increases if x > 0* | *Decreases* | *Increases if x > b/a* |
