# Supplementary material for: Within-Host Dynamics of Multi-Species Infections: Facilitation, Competition and Virulence
Source: PLoS One. 2012 Jun 21;7(6):e38730. doi: 10.1371/journal.pone.0038730 (PMC3380906; doi:10.1371/journal.pone.0038730)
Supplement: Text S1 — Model Equilibria and Stability Analysis. (DOC) [file pone.0038730.s003.doc]

**Text S1.**

**Model Equilibria and Stability Analysis**

We model the dynamics of two parasite species, A and B, within a single host, using the simple two-species competitive Lotka-Volterra equations:

*dA/dt = A (1- A - xB)*

*dB/dt = B (1- yA - zB)* [1]

*x* and *y* are the interspecific competition coefficients of B on A, and A on B, respectively. *z* is the intraspecific competition coefficient of B (i.e. B self-competition) and this implies that the carrying capacity of B is 1/*z* times the carrying capacity of A (where *z* > 0).

The model has three equilibria:

- A alone at *A* = 1;*
- B alone at *B* = 1/z;*
- Coexistence at *A* = (x - z) / (xy - z)*, *B*= (y - 1) / (xy - z).*

A stability analysis of model 1 reveals that when *y* > 1 (i.e. when A is more inhibitory to B than to itself) then pure A is locally stable. Similarly, if *x* > *z* (i.e. when B is more inhibitory to A than to itself), then pure B is locally stable. This suggests that when conditions for pure A and pure B are both satisfied, the system is bistable. Whether the system approaches a pure A or pure B equilibrium will depend on initial conditions, with the most abundant (i.e., resident) population typically having a decisive advantage. The repellor (*p**), which is the proportion threshold of parasite A invasion, is defined by *p** = *A* / (A* + B*)* = (*z - x*) */* (*z - x - y + 1*). When the proportion of parasite A exceeds *p**, parasite A invades and go to fixation; otherwise parasite B invades and go to fixation (Fig. S1A). Also, the threshold of A invasion (*p**) increases with increasing B inhibition to A (*x*) whereas it decreases with increasing A inhibition to B (*y*) (Fig. S1B), and decreases with increasing B self-inhibition (*z*) (Fig. S1C).

The stability analysis of the coexistence equilibrium (A*, B*) reveals that the equilibrium is stable if *xy* < *z*, *y* < 1 and *x* < *z* (note that we assume *z* > 0). Therefore, one-way interspecific competition (i.e. either *x* > 0 and *y* ≤ 0, or *y* > 0 and *x* ≤ 0) is stable whenever *y* < 1 and *x* < *z*. Reciprocal facilitation (i.e. both *x* and *y* negative) is stable whenever *xy* < *z*. This means that for sufficiently strong reciprocal facilitation (*xy* > *z*) all the equilibria (pure A*, pure B* and coexistence) are destabilized and the within-host dynamics enter into a runaway process (Fig.1, red region). Reciprocal interspecific competition (i.e. both *x* and *y* positive) is stable if the conditions for pure A* and pure B* stability do not hold.

**Effect of changing *x*, *y*, and *z*, on equilibrium densities of parasite A (*A**), parasite B (*B**), and on total virulence (*V**)**

Our analytical results (summarized on Table S1) reveal that the density of *A** always decreases with increasing values of B inhibition to A (*x*). *A** increases its density by increasing its inhibition to B (*y*) if B inhibits A (*x > 0*) (i.e. by increasing the harm to a competitor) and if B inhibition to A is lower than B self-inhibition (*z*), otherwise B invades. *A** increases with *z* if B inhibits A (*x > 0*) and *y* is lower than 1 (i.e. A’s interspecific competition on B is lower than A’s intraspecific competition). Thus if B’s self-inhibition increases, A only receives an advantage if B inhibits A. This suggests that if B helps A (*x* < 0), increasing B intraspecific competition (*z*) is disadvantageous to A. The density of *B** always decreases with increasing values of A’s inhibition to B (*y*), and intraspecific competition (z). The density of *B** may increase with *x* if A’s interspecific competition on B (*y*) is lower than A’s intraspecific competition (i.e. *y* < 1), otherwise A invades.
